# Bone Tissue Engineering

**DOI:** 10.1007/s40610-015-0022-2

**Published:** 2015-08-15

**Authors:** Cameron R. M. Black, Vitali Goriainov, David Gibbs, Janos Kanczler, Rahul S. Tare, Richard O. C. Oreffo

**Affiliations:** Bone and Joint Research Group, Centre for Human Development, Stem Cells and Regeneration, Developmental Origins of Health and Disease, Institute of Developmental Sciences, University of Southampton Medical School, Southampton, SO16 6YD UK

**Keywords:** Skeletal stem cell, Osteoprogenitor, Biomaterial, Osteogenesis, Translational research, Bone tissue regeneration

## Abstract

Medical advances have led to a welcome increase in life expectancy. However, accompanying longevity introduces new challenges: increases in age-related diseases and associated reductions in quality of life. The loss of skeletal tissue that can accompany trauma, injury, disease or advancing years can result in significant morbidity and significant socio-economic cost and emphasise the need for new, more reliable skeletal regeneration strategies. To address the unmet need for bone augmentation, tissue engineering and regenerative medicine have come to the fore in recent years with new approaches for de novo skeletal tissue formation. Typically, these approaches seek to harness stem cells, innovative scaffolds and biological factors that promise enhanced and more reliable bone formation strategies to improve the quality of life for many. This review provides an overview of recent developments in bone tissue engineering focusing on skeletal stem cells, vascular development, bone formation and the translation from preclinical in vivo models to clinical delivery.

## Introduction

The loss or dysfunction of skeletal tissue that can accompany trauma, injury, disease or advancing years can result in significant morbidity as well as a variety of socio-economic issues. In addition, changing patient demographics, together with rising patient expectations and increasing complexity of ensuing clinical scenarios, provide the imperative for new, more reliable skeletal regeneration strategies. Current approaches to replace or restore significant quantities of lost skeletal tissue come with substantial limitations and inherent disadvantages that may be harmful. Tissue engineering and regenerative medicine have come to the fore in recent years with new approaches for de novo skeletal tissue formation in an attempt to address the unmet need for bone augmentation and skeletal repair. These approaches seek to harness stem cells, innovative scaffolds and biological factors to create, ideally, robust, reproducible and enhanced bone formation strategies to improve the quality of life for an ageing population. The following reviews recent developments in bone tissue engineering and regeneration, focusing on skeletal stem cells, vascular development and bone formation. In addition, we review current developments in the translation from preclinical in vivo models to clinical delivery. We detail current regenerative strategies in development to augment bone formation in a range of orthopaedic scenarios including examination of some of the translational issues before patient treatment, much heralded, can be routinely achieved.

## Skeletal Stem Cells

The remarkable ability of bone to remodel, coupled with the capacity of bone tissue to heal/regenerate following fracture, supports the concept of a stem cell population within postnatal bone marrow. Typically, bone marrow comprises the stromal and haematopoietic compartments, and the existence of a haematopoietic stem cell (HSC) population has long been acknowledged. Bone marrow cell suspensions include both haematopoietic cells and non-haematopoietic stromal cells. The stromal tissue functions as a scaffold, composed of a network of cells that provide physical and functional support to the haematopoietic cells. The stromal fraction, characteristically, are able to adhere to tissue culture plastic, while the non-adherent haematopoietic cells can be readily removed from the adherent stromal cell cultures by a simple wash step.

Seminal experiments by Alexander Friedenstein and co-workers were instrumental in demonstrating that a culture of bone marrow stromal cells, at clonal density, enabled identification of cells, referred to as colony-forming units-fibroblastic (CFU-Fs), which were capable of establishing clonal growth in a density-independent fashion [1]. Furthermore, Friedenstein and colleagues established unequivocally that bone marrow stromal tissue and the derived clonogenic fraction could generate bone following in vivo transplantation. Since multiple tissues, such as bone, cartilage and fat, were identified in heterotopic transplants of cell populations originating from a single stromal cell, it was concluded that osteogenic, chondrogenic and adipogenic phenotypes were part of a multilineage system downstream of the common progenitor cell population retained in postnatal bone marrow stroma. This multipotent stromal progenitor cell population constituted a second type of stem cell population, in addition to HSCs, within bone marrow referred to as the osteogenic (Friedenstein) or stromal (Owen and Friedenstein) or mesenchymal (Caplan and Pittenger) or skeletal (Bianco and Robey) stem cell population [1, 2].

An array of cell-surface antigens, namely the trypsin-resistant antigen recognised by the STRO-1 antibody, CD29 (β1 subunit of integrin family), CD44 (glycoprotein expressed by both HSCs and mesenchymal stem cells (MSCs)), CD49a (laminin and collagen receptor VLA-α1), CD73 (ecto-5′-nucleotidase), CD90 (Thy-1), CD105 (Endoglin), CD106 (VCAM-1), CD146 (MCAM), CD166 (ALCAM) and CD271 (low-affinity nerve growth factor receptor), are expressed by skeletal stem cells (SSCs). These markers have been used to enrich for the skeletal stem cells from a heterogeneous bone marrow mononuclear cell fraction using a positive immunoselection strategy [3•]. Alternatively, SSC enrichment can be achieved by negative immunoselection for haematopoietic cell-surface antigens such as CD34, CD45, CD19, CD14, CD11b, CD79α and HLA-DR surface molecules [3•]. However, the lack of consensus on the exact nature of cell-surface marker/s unique to SSCs, a spectrum of ‘stemness’ within the SSC population due to variable expression of certain markers and the inherent variation in biological systems highlight the difficulty in defining a unique SSC signature. Thus, the absence of a specific SSC marker has contributed, in part, to conflicting data within the literature on the characteristics of the SSC.

A widely accepted perspective, championed by Paolo Bianco and co-workers, advocates that the SSC population is a precisely defined physical and conceptual entity, which resides in a subendothelial position in the perivascular spaces surrounding the vascular sinusoids/distinctive venous vessels occurring in bone marrow [4••]. The researchers observed that, upon transplantation in vivo, the SSC was capable of generating a complete heterotopic bone or bone marrow organ/ossicle (including a compartment of perivascular stromal cells with similar phenotypes and properties as the originally explanted cell) in which haematopoiesis from the recipient animal was established. Thus, as demonstrated by Bianco and co-workers, the ability to establish, organise and transfer the haematopoietic niche in vivo is an important defining feature of SSCs. In contrast, a view proposed by Mark Pittenger and Arnold Caplan characterises MSCs as products of their environment, and it is possible to develop this environment in vitro [5]. Thus, in accordance with this view, MSCs can adopt two phenotypes: (i) a ‘constitutive’ phenotype in which, as perivascular cells, the population expresses the characteristic cell-surface markers both in vivo and ex vivo and demonstrates functionality through its multipotential ex vivo differentiation capabilities, and (ii) a ‘regulatory’ phenotype in situations of tissue/vessel damage in vivo, where the released pericytes are activated by injury to become MSCs that then serve as site-regulated, multidrug dispensaries to promote and support the natural regeneration of focal injuries [6•].

The pleiotropic effects of SSCs on cells of the immune system have been extensively detailed in the literature, and SSCs are often regarded as immune privileged or non-immunogenic, given their phenotype is widely described as MHC class I+, MHC class II−, CD40−, CD80− and CD86− [6•, 7–10]. However, before the full therapeutic potential of this adult stem cell population can be realised, rigorous studies in defined in vivo systems are needed to elucidate the precise identity of the regulatory effects of SSCs on immune and inflammatory cells. Critically, the successful application of SSCs in bone regeneration strategies necessitates the development of simple, safe and efficacious culture techniques that allow ex vivo expansion of SSCs without loss of their functional, immunophenotypic and cytogenetic characteristics. Development of such protocols in combination with strategies for robust osteogenic differentiation of human SSCs (typically, through the application of factors, hormones, supplements) and incorporation of smart matrices/scaffolds auger well for bone tissue formation [11].

## Scaffold Matrices for Bone Regeneration

The concept of a biomaterial has been defined as ‘a substance that has been engineered to take a form which is used to direct, by control of interactions with components of living systems, the course of any therapeutic or diagnostic procedure’ [12]. The scaffold provides the extracellular microenvironment for the support and stimulation of stem/cell-driven tissue regeneration serving as a supportive platform for transplanted cells or recruiting and retaining endogenous cells together with appropriate mechanical cues and biological triggers. Approaches in biomaterials are driven by a desire to replace the structural aspect of a diseased tissue or organ and to trigger/harness regenerative processes capable of restoration of functional biology. To perform such functions, biofunctionalisation and tissue integration together with appropriate degradation characteristics in the absence of any significant adverse reactions are important attributes in biomaterial development for clinical application.

A wealth of materials are in development and indeed already exist that seek to incorporate physical, chemical and biological signalling cues, to create appropriate regenerative host microenvironments, in an attempt to aid the healing/regenerative processes. These include scaffolds such as (i) *bioceramics*—incorporating hydroxyapatite or calcium phosphates that typically exhibit good bone integration, are osteoconductive and display a high compressive strength; (ii) *natural polymers*, such as extracellular matrix proteins (e.g. collagens, fibrin, elastin, alginate, hyaluronic acid) and xenogeneic derived materials, are intrinsically biocompatible and have reached clinical use with minimal adverse immunological reports; (iii) *synthetic polymers—*a variety of macromolecules (e.g. polyethylene glycol (PEG)) modulated on the basis of their monomer constituents, the relative ratios of co-polymers and the interactions/functionalisation of polymer side chains creating a raft of materials for tissue engineering; and (iv) *hydrogels—*hydrated polymer chains, offer significant potential in the delivery of cells and growth factors [13]. These materials can typically support the adhesion of cells and other ECM proteins, enable migration of cells and facilitate incorporation of bioactive molecules and nutrients and their subsequent controlled, in time and space, targeted release. In recent studies from Dawson et al., a synthetic clay hydrogel was shown to be suitable for delivery through injection (with subsequent gel network self-reassembly) of growth factors and cells and to be capable of stimulating in vivo angiogenesis [14••].

An area that has seen significant development is additive manufacturing (AM), the computer-directed process of 3D layer-by-layer model fabrication [15]. AM offers the potential for fabrication of implants of exquisite complexity that could range from permanent to biodegradable, with a potential intermediate ability to be incorporated into the host bone tissue. Despite recent advances, the usual issues/challenges, as for all current large material constructs of vascularisation, integration and, to date, economic viability remain. Thus, while current technological developments in the biomaterials field create an exciting environment enabling further rapid progress, clinical translation of experimental findings remains limited.

## Development of Vascularised Bone

Establishment of a blood vessel network by vasculogenesis and angiogenesis is essential for cellular gaseous exchange, nutrient supply and waste product removal during new tissue formation. There is significant emerging evidence that blood vessels act as a reservoir for an undifferentiated skeletal progenitor that translocates with new vessel ingrowth to areas of tissue damage and repair. Furthermore, the interaction between inflammatory cues, cellular components and vascular elements is a highly intricate, spatiotemporal event essential in bone tissue repair. It is therefore not surprising that the complex biochemical and physical associations between skeletal cells and neighbouring blood vessels have been identified as an essential requisite for dynamic bone development and repair. Thus, insufficient or incongruous vascularisation of bone will ultimately result in irregular bone formation, delayed union or non-union of bone fractures [16, 17]. Hence, the regeneration of a functional vascular supply concomitantly with bone osteogenesis is central to the regeneration of functional bone in, for example, critical-sized skeletal defects.

The essential molecular crosstalk between endothelial cells and skeletal progenitor cells in osteogenesis have been investigated using pharmacological, molecular and biochemical studies to identify the factors that are the predominant players in this coupling [16, 17]. These include vascular endothelial growth factor (VEGF), fibroblast growth factor (FGF), placental growth factor (PGF), the angiopoietins (ANG 1 and 2), transforming growth factor (TGF) superfamily (BMP-2, BMP-7, BMP-9, TGF-β), receptor activator of nuclear factor kappa ligand (RANKL), biglycans and endothelin-1 [16, 17]. Furthermore, recent studies have identified microRNAs to affect fracture repair. As well as the predicted and targeted cytokines and growth factors, modulation of tissue and cellular oxygen gradients has emerged as key determinants in angiogenesis resulting in profound effects on bone. Furthermore, inhibition of VEGF receptors on skeletal progenitor populations (osterix-CRE) has been implicated in the reduction of trabecular bone [16, 17].

The vasculature has come to the fore in bone biology in recent years with the concept advanced and evidenced that the pericyte cell (encompass microvessels or vessel adventitial cells) is the potential precursor for the skeletal stem cell, central in bone repair [18]. Furthermore, bone stem cells of the bone marrow have been found to reside in the perivasculature and the detection of these cells using the nestin-GFP transgene demonstrated expression of both pericyte markers alpha smooth muscle actin (αSMA) and NG2. Mediators such as DJ-1 (a 189-amino acid protein encoded by PARK7) which modulates FGF receptor 1 signalling activation and glycoprotein non-metastatic melanoma protein B (GPNMB) have been identified in the complex cross communication between vascular and osteogenic cells during bone regeneration. Notch signalling has now been shown to induce proliferation of endothelial cells in postnatal long bone, and interestingly, the disruption of Notch signalling in a specific sub-population of endothelial cells affected skeletal bone properties together with impaired angiogenesis [19, 20••].

There is no doubt therapeutic targeting of angiogenesis to treat non-healing bone defects will be essential for improved clinical skeletal outcomes. Treatments such as erythropoietin (EPO) have been used to provide a good effect given the potential of EPO to induce cartilaginous callus formation and angiogenesis resulting in enhanced endochondral ossification. Vascular factors are also integral in other skeletal repair targets such as distraction osteogenesis or the repair of hypoplastic facial bone defects. Thus, key is the development of the appropriate balance of bone and blood vessel integration in what are tightly temporally and spatially regulated systems. Creation of bioengineered heterotopic bone around new blood vessels as suggested by Cai and colleagues and altering the levels of growth factors such as VEGF and BMP-2 to modulate bone repair [21•] is an interesting suggestion to enhance bone augmentation. Undoubtedly, the development of new vascular ingrowth therapies will dramatically help in the application of bone cellular constructs in augmenting bone repair.

## Development of In Vivo Models of Bone Repair

A wealth of in vitro data over the last four decades has elucidated invaluable information on the molecular and cellular mechanisms involved in osteogenic repair, and the more recent development of complex, multicellular, three-dimensional models has significantly enhanced our understanding of osteogenesis and bone healing. However, these techniques remain unable to mimic the cellular, molecular, physiological and biomechanical intricacies present at the whole organism level. Critical aspects in bone repair such as the presence of a patent vascular network and biomechanical stimulation have proven difficult to reproduce outside of the living organism and to date necessitate the application appropriate in vivo approaches. Similarly, a number of factors influence appropriate animal use and in vivo design (Table 1), and thus, the translational development of novel therapies in bone repair and fracture healing requires, typically a continuum of in vivo modelling, progressing through initial feasibility studies into clinically relevant translational models.

**Table 1:** Selection and design of in vivo animal models

In vivo species and breed selection criteria	Considerations for in vivo model design
• Size and anatomical characteristics • Biomechanical characteristics • Physiological similarity to humans • Gene sequence and protein homology to humans • Genetic variability between cohorts • Availability • Husbandry and handling, risk to staff • Housing requirements • Resistance to disease and environmental stress • Lifespan and life-stage progression throughout study duration • Existence of anaesthetic, surgical and post-operative protocols • Robust nature, tolerant of experimental model • Surgical survivability • Ethical and societal implications	• Model design addresses the research question • Develop model to improve data relevance • Modification to eliminate procedural shortcomings of previous techniques to address shortcomings • Occur after completed in vitro toxicity, cytocompatibility and efficacy assays • In vitro feasibility justifies use of animal model • Study group design, including appropriate controls • Adequate group numbers, avoiding excessive animal use • Avoid wasteful experimental duplication • Impact of model on animal behaviour • Model design closely simulates possible clinical usage • Consider methods of post-experimental analysis

Reconstitution of the multifaceted and complex system involved in bone repair in an experimental in vivo environment needs to ensure inclusive examination of all processes involved. Table 2 summarises common in vivo skeletal models, a number can be seen to overlap, while ‘mechanistic’ models detail in vivo models that aim to simulate clinical application representing end-user specific systems in translational research.

**Table 2:** Summary of common animal models in musculoskeletal research and systems assessed

System assessed	Model description	Common species	Relevant publications
Vascular	Chorioallantoic membrane (CAM) model, sub-cutaneous and intramuscular implantation	Chick egg and embryo, mouse, rat, rabbit, humanised animal models	Nowak-Sliwinska, P et al. 2014 [22] Smith, EL et al. 2013 [23••]
Intramembranous ossification	Partial and full thickness cranial defects, uni-cortical long bone defects, hip and sternal defects	Mouse, rat, rabbit, goat, pig, sheep	Yamano, S et al. 2014 [24]
Endochondral ossification	Ectopic models, sub-cutaneous and intramuscular implantation, long bone defect models	Mouse, rat, rabbit, dog, goat, mini-pig, pig, sheep, primate	Shim, JH et al. 2014 [25] Berner et al. 2013 [26] Suarez-Gonzalez, D et al. 2014 [27]
Mechanistic	Spinal Fusion, dental implantation, distraction osteogenesis, orthopaedic and dental device implants, metabolic, neoplastic and infectious disease models, molecular targeted healing	Rat, rabbit, dog, goat, mini-pig, pig, sheep, cow and horse, primate, human	Ke, HZ et al. 2012 [28] Monroea, D et al. 2012 [29] Oheim, R et al. 2013 [30•] Brodano, GB et al. 2014 [31]

Relevant publications have been selected to introduce the most recent advances in surgical design, scaffold production, factor delivery and molecular intervention, comprehensively reviewed in Gothard, D et al. [32•]

## Small Animal Models

Small animal preclinical models of bone repair have proved efficacious in the assessment of biomaterial and cellular performance providing information on localised and systemic toxicity, tumourigenesis, cytological and biological compatibility, vascular integration and potentially deleterious off-target effects. Models include the application of complete and partial osteotomies of long bones, calvaria, sternum and hip bones as well as sub-cutaneous and intramuscular implantation. The use of mouse, rat and rabbit animal models, relatively accessible to the researcher, allows analysis of bone regeneration and repair in relatively short time frames and with, as required, robust animal numbers. The sub-cutaneous model involves a simple full thickness skin incision under aseptic conditions and subsequent evaluation of a number of implants in a single animal providing data within a period of time varying from days to months on vascular compatibility and bone regeneration simultaneously using contrast-enhanced micro-computed tomography (μ-CT) supplemented by traditional histological techniques. The ectopic nature of this model limits evaluation of bone augmentation in a native bone environment and necessitates the development of bone-specific models such as the calvarial and femoral segmental models. While small animal models are cost effective, accessible and offer useful information on biological performance, such approaches are restricted by the scale and limited clinical relevance and, therefore, a need for large animal models.

## Large Animal Models

The design of large animal models typically develops from either a scaled up small animal model or adaptation of a model based on a human clinical need. The former can be exemplified by long- and flat-bone defects with or without a form of mechanical fixation. The latter are as variable as the clinical targets and typically utilise species of comparable human biomechanical and physiological composition, predominantly in the pig and sheep (comprehensively reviewed by Gothard et al. [32•]). Large animal models allow the assessment of large volume repair over a longer time frame sufficient to examine remodelling and implant integration. Models such as spinal fusion, hip/knee arthroplasty and fixator development are clinically analogous and therefore offer insight into the development of treatment options. The advent of new emerging therapies, such as growth factor and small osteogenic molecule delivery, are an exciting move towards minimally invasive treatment options of bone disease and repair. As treatment modalities encompass not only engineered scaffolds and implants but also biochemical and molecular interventions, the match/mismatch between animal and human physiology will remain central with variations in gene and protein sequence homology presenting the potential for misrepresentative data from in vivo models. Conversely, the wealth of genetic and therapeutic data from small animal studies offers exciting new avenues when harnessed with cell- and scaffold-based modalities for bone augmentation.

## Clinical Translation

In harnessing regenerative and tissue engineering strategies, the requirements of a tissue engineering solution are very much dependent upon the clinical scenario. A clinical issue such as tibial atrophic non-union may require only stimulation of fracture healing, whereas other bone repair situations may require merely mechanical support. In contrast, segmental bone defects typically require the provision of vascularised bone with the ability to integrate to the surrounding tissues and thus present a significant challenge. The following highlight relatively recent developments on the application of tissue engineering strategies in the management of fractures, arthrodesis, osteochondral defects and segmental bone defects.

### Fractures and Arthrodesis

Over a decade ago, BMP was delivered using a collagen sponge within a cage device (Infuse, Medtronic) or collagen putty (OP-1, Stryker) to mediate spinal arthrodesis and fracture healing. While initial results were promising, subsequent cases and clinical reports have demonstrated that the high doses of BMP used together with poor growth factor localisation culminated in osteolysis and heterotopic ossification [33]. Furthermore, to date, there remains controversy as to the clinical effectiveness of BMP in the treatment of open tibial fractures [34]. Ceramic bone graft, when used in combination with fresh autologous bone marrow aspirate, has been shown to be safe and as effective as autologous bone grafting (ABG) in stimulating some forms of spinal fusion [35, 36]. Interestingly, while the addition of an osteoinductive material appears to be a prerequisite for spinal fusion, there is emergent data demonstrating calcium phosphate cement application could produce outcomes equivalent to ABG in the management of tibial plateau fractures [37]. Furthermore, a case series has shown that ceramic graft use in impaction bone grafting of the acetabulum produced good medium-term results. In support of such observations, Damron and colleagues have shown the addition of bone marrow aspirate to ceramic graft applied to treat benign bone cavity defects provided no added advantage (Damron et al.) [38] with ceramic bone alone sufficient to produce healing.

### Osteochondral Defects

Osteochondral defects in the knee are typically managed through the application of microfracture, or autologous chondrocyte implantation, a two-stage surgical procedure [39]. Synthetic multiphasic scaffolds, without additional cells or growth factors, have been used clinically to treat osteochondral defects; however, their efficacy remains to be demonstrated in a randomised controlled trial [40]. An alternative therapy has been developed in which autologous articular chondrocytes are harvested, seeded upon a synthetic scaffold material and applied to the defect in a single procedure [41]. Initial results appear promising; however, while this technique avoids a second surgical procedure, it does involve articular chondrocyte harvest, and to date, the long-term outcome remains unknown. Initial results appear promising; however, while this technique avoids a second surgical procedure, the approach involves articular chondrocyte harvest, and to date, the long-term outcome remains unknown. Areas of current exploration for osteochondral defect treatment include application of chondroprogenitor cells from retro patella or sub-cutaneous adipose tissue in combination with printed scaffolds generated using additive manufacturing in a single surgical procedure [15].

### Bone Defects

**Fig. 1:**
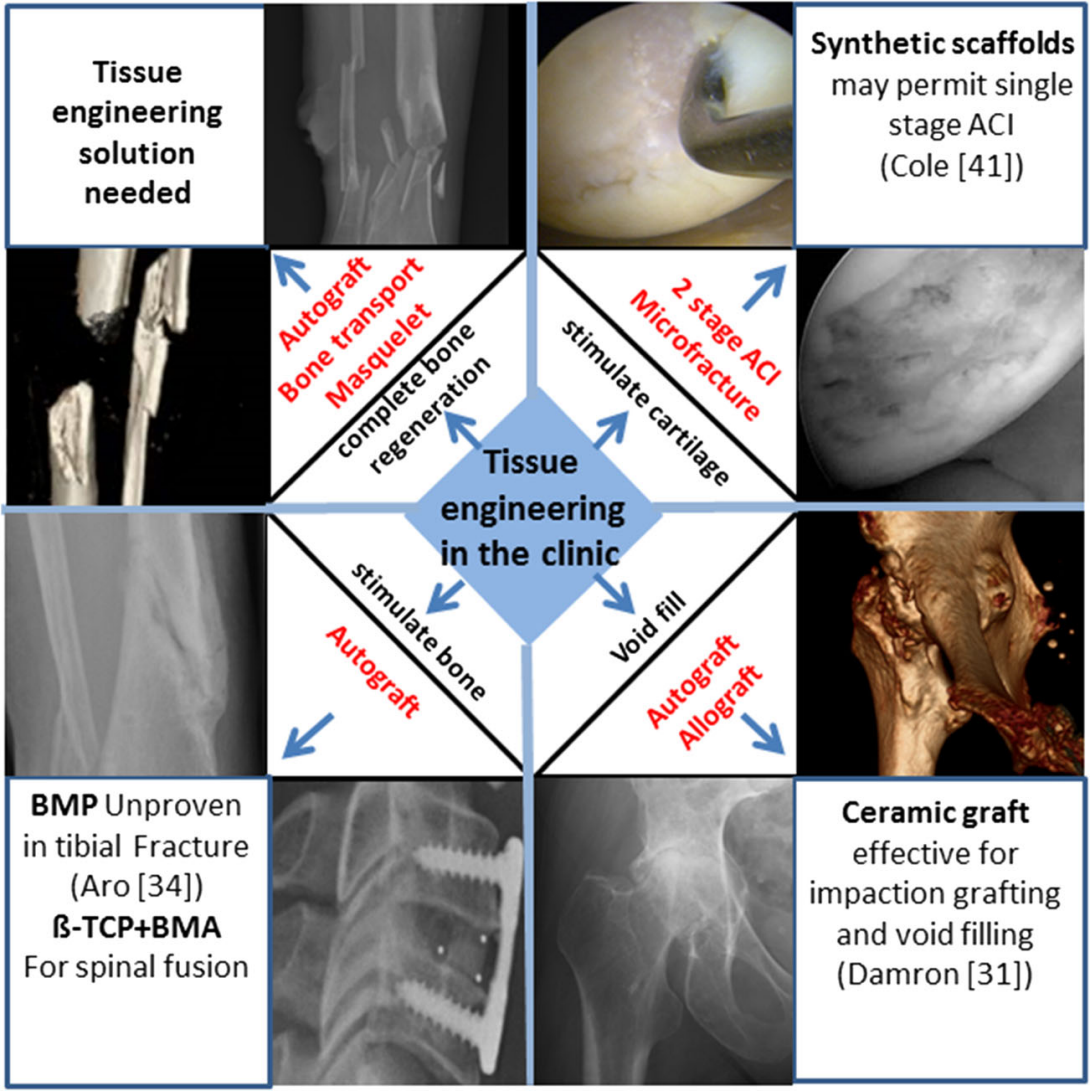
Skeletal tissue engineering in the clinic. Clinical skeletal repair requirements: (i) cartilage regeneration, (ii) bone void filling, (iii) stimulation of fracture healing or arthrodesis and (iv) reconstruction of segmental bone loss are represented in different quadrants. (i) In the *top right quadrant* are arthroscopic images of an osteochondral lesion (*upper*) and chondral lesion post microfracture (*lower*). (ii) In the *lower right quadrant* is a 3D reconstruction (*upper*) and radiograph (*lower*) of a patient with severe osteoarthritis and protrusio acetabuli. (iii) In the *lower left quadrant* show radiographs demonstrating fracture non-union and spinal arthrodesis. (iv) In the *top left quadrant* demonstrates a comminuted tibial fracture (*upper*) and segmental bone defect (*lower*). Current treatment strategies are detailed (in *red*) in corresponding *triangles*

Segmental bone defects are most commonly found in the tibia and result in amputation if structural integrity cannot be restored and maintained. Current treatment strategies include the application of (i) isolated ABG, (ii) vascularised ABG, (iii) induced membrane technique (Masquelet) with ABG and (iv) bone transport procedures. Karger and colleagues and Meinig and co-workers have combined synthetic bone and membrane material with ABG in post-traumatic and bone defect reconstructions using the induced membrane technique [42, 43]. BMP-2 combined with allograft has been shown to be as effective as ABG in the management of diaphyseal tibial cortical defects [44]. Current studies include application of stem-progenitor cell-seeded scaffolds [45•] and variations on the Masquelet technique using a synthetic bioabsorbable angiogenic and osteogenic membrane [46] with synthetic and autograft materials. To date, the development of a mechanically robust, vascularised bone construct providing an integrated tissue engineering solution for segmental bone defects remains to be clinically translated (Fig. 1).

The use of cell-based therapies in skeletal regenerative medicine is an area of current intense research focus. The efficacy of current approaches has yielded ambiguous results with limited consensus on the most effective cell origin type, number, combination and method of delivery. In the majority of current experimental models, the introduction of a cell progenitor source, alone or in combination with biomaterials, typically has occurred at the time of wound creation, with cells immediately localised in vivo into an acute inflammatory physiological environment. During the initial phases of tissue trauma, the affected tissue is rich in cyto-modulatory peptides including TNF-α, interleukins and interferons as well as raised serum acute-phase protein concentrations. Typically, within the tissue engineering/regenerative community, it has been thought that the effectiveness of stem and progenitor populations introduced into such a perceived hostile inflammatory milieu is inhibitory/detrimental to tissue reparation. However, recent studies using spatiotemporal manipulation of cell delivery, specifically, delayed injection of cells into a wound site, after the acute inflammatory response subsides has generated exciting results experimentally in the fields of segmental bone tissue engineering [47•], cardiology [48] and neuronal repair [49]. Delayed injection experimental models utilising readily accessible adult-derived bone marrow stromal cells have shown enhanced repair of damaged tissue compared to ‘time-of-trauma’ cell applications. It is evident that the means and timing of cell delivery influence treatment efficacy. Enhanced reparation is observed when cells are introduced sub-cutaneously, intravenously as well as directly to the wound site [47•, 48–50]. Critically, the advantages noted experimentally have been realised in orthopaedics [51] and prospectively offer effective advanced cell-based treatment modalities in regenerative medicine

## Future Perspectives

To date, SSC therapy is hampered predominantly by our limited understanding of skeletal stem cell fate, immuno-phenotype and selection criteria. There is a need for facile, safe and efficacious protocols of stem cell isolation and expansion together with enhanced bioinformatics knowledge on the phenotypic ‘fingerprint’ of the skeletal stem cell at a single-cell resolution and the generation of skeletal cells from pluripotent stem cell sources. It is likely new cell approaches and the development of ‘smart’ hydrogels, able to temporally and spatially control growth factor release to render safe and efficacious growth factor use in stimulation of fracture healing and arthrodesis, are areas that will see significant development. The next 5 to 10 years will see intense interest in the potential of additive manufacture to produce synthetic multiphasic scaffolds in which the internal architecture and topography are analysed for cartilage and bone regeneration requirements. It is likely approaches will include the development and integration of immuno-privileged constructs containing an appropriate scaffold/growth factor(s) composition for autologous and, potentially, allogeneic skeletal populations. For routine and, critically, robust bone formation approaches, the future challenge for regenerative medicine will be the development of bioengineered vascularised grafts at appropriate scale for clinical application.

## Conclusions

The relative accessibility of an autologous osteoprogenitor population has driven the application of skeletal stem cell therapy for orthopaedic application in contrast to other stem cell sources (pluripotent embryonic stem cells or adult stem cell populations from connective tissues).

For cell-based clinical application of skeletal populations to be realised, simple and robust isolation together with safe and efficacious delivery approaches as well as defined in vivo systems will be key that ensure maintenance of cell function, immuno-phenotype and cytogenetic characteristics. Work continues apace in a number of groups to generate matrices that will permit temporal-spatial growth factor release with seeded stem cells to promote prevascularised prior to application as well as vascularised bone constructs. While the treatment of large segmental bone defects remains a significant challenge with successful repair or the integration of large regenerative tissue constructs critically dependent on development of a functioning blood supply, nevertheless, these are exciting times in skeletal regenerative medicine with significant new approaches emerging for bone repair for an increasing ageing demographic.
